# Atrial Fibrillation Ablation in a Patient with Cor Triatriatum Sinister and Left Common Pulmonary Vein: Impact of Left Atrium Anatomy on Ablation Approach

**DOI:** 10.3390/life12070992

**Published:** 2022-07-04

**Authors:** Ioan-Alexandru Minciună, Gabriel Cismaru, Mihai Puiu, Radu Roșu, Denis Amet, Daniela Anghelina, Alexandra Gica, Raluca Tomoaia, Marius Andronache, Dana Pop

**Affiliations:** 1 5th Department of Internal Medicine, Faculty of Medicine, “Iuliu Hațieganu” University of Medicine and Pharmacy, 400012 Cluj-Napoca, Romania; minciuna.ioan.alexandru@elearn.umfcluj.ro (I.-A.M.); ovidiu.rosu@umfcluj.ro (R.R.); raluca.tomoaia@elearn.umfcluj.ro (R.T.); dana.pop@umfcluj.ro (D.P.); 2 Department of Cardiology, Clinical Rehabilitation Hospital, 400347 Cluj-Napoca, Romania; puiu.mihai@yahoo.com; 3 Adult Congenital Heart Disease Medico-Surgical Unit, AP-HP, Georges Pompidou European Hospital, 75015 Paris, France; denis.amet@egp.aphp.fr; 4 Cardiology Department, AP-HP Paris-Saclay, Bicêtre Hospital, 94270 Le Kremlin-Bicêtre, France; daniela.anghelina@aphp.fr; 5 Department of Cardiology, Ares Hospital, 400015 Cluj-Napoca, Romania; alexandragica@monza-ares.ro; 6 Cardiology Department, CHU Clermont-Ferrand, Clermont University, ISIT-CaVITI, 58 rue Montalembert, 63000 Clermont-Ferrand, France; marius.andronache@chu-clermontferrand.fr

**Keywords:** cor triatriatum sinister, atrial fibrillation, catheter ablation, left common pulmonary vein, high-power, short-duration ablation

## Abstract

Atrial fibrillation is the most common presentation in adult patients with cor triatriatum sinister. The key to successful and safe catheter ablation in these patients is an accurate exploration and thorough understanding of the left atrial anatomy, both before and during the procedure. Catheter manipulation is highly dependable on left atrial anatomy, including the interatrial septum, insertion of pulmonary veins and cor triatriatum membrane. Anatomical variants such as the left common pulmonary trunk may influence the ablation approach and outcome. We report the case of a 52-year-old patient with cor triatriatum sinister and the left common pulmonary vein variant who underwent successful high-power, short-duration catheter ablation for paroxysmal atrial fibrillation.

## 1. Introduction

Cor triatriatum is a rare congenital cardiac anomaly that accounts for 0.1 to 0.4% of all congenital heart disease [1,2,3,4,5]. The left-sided anomaly cor triatriatum sinister (CTS) is more frequent than the right-sided cor triatriatum dexter, with both conditions being commonly diagnosed in infancy and associated with other congenital heart defects such as atrial septal defect, patent foramen ovale or left superior vena cava [1,2].

In CTS, abnormal fibromuscular septation divides the left atrium into two separate chambers: the pulmonary chamber, usually located posteriorly and superiorly, which most frequently receives the four pulmonary veins, and the true left atrium, located anteriorly and inferiorly, which contains the left atrial appendage and the mitral valve annulus [1,2,3,4,5,6].

CTS is an uncommon diagnosis in adulthood, with the symptoms depending on the degree of hemodynamic obstruction between the two left atrial chambers. As a consequence of this, adult patients with CTS and large non-obstructive fenestrations in the membrane may present as completely asymptomatic, whereas patients with small obstructive fenestrations may mimic the clinical picture of mitral stenosis due to the obstruction through the membrane [1,2,6]. However, although it is still not completely understood why asymptomatic patients with large fenestrations become symptomatic with age, in most of them, atrial fibrillation is the sentinel clinical symptom [2,5,6,7].

AF catheter ablation may be considered in patients with congenital heart defects, as the current practical guidelines recommend [8]. Nevertheless, in patients with CTS, catheter manipulation is highly dependent on left atrial anatomy, including the anatomy of the interatrial septum, membrane and pulmonary veins [4,6,9]. Although the left common pulmonary vein is a common anatomical variant in patients undergoing AF catheter ablation [10,11], there are no reports of this particular anatomy in patients with CTS. We report the case of a patient with CTS and the left common pulmonary vein variant who underwent successful high-power, short-duration catheter ablation for paroxysmal AF.

## 2. Case Presentation

A 52-year-old Caucasian patient was referred to our arrhythmias department for recurrent paroxysmal AF episodes, which began three months earlier, in order to assess their suitability for catheter ablation. He had no medical history or past interventions and at admission was on antiarrhythmic treatment with Flecainide and Bisoprolol, which had commenced with the onset of symptoms. The patient reported worsening symptoms in the preceding two weeks, with AF episodes becoming more frequent, lasting longer and affecting his everyday activity.

**Figure 1:**
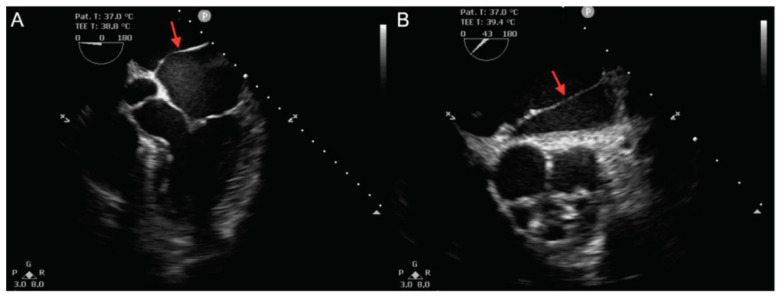
Transesophageal echocardiography. (**A**): Mid-esophageal 5-chamber view and (**B**): Mid-esophageal short-axis view, showing a membrane (red arrows) separating the left atrium in two separate chambers.

**Figure 2:**
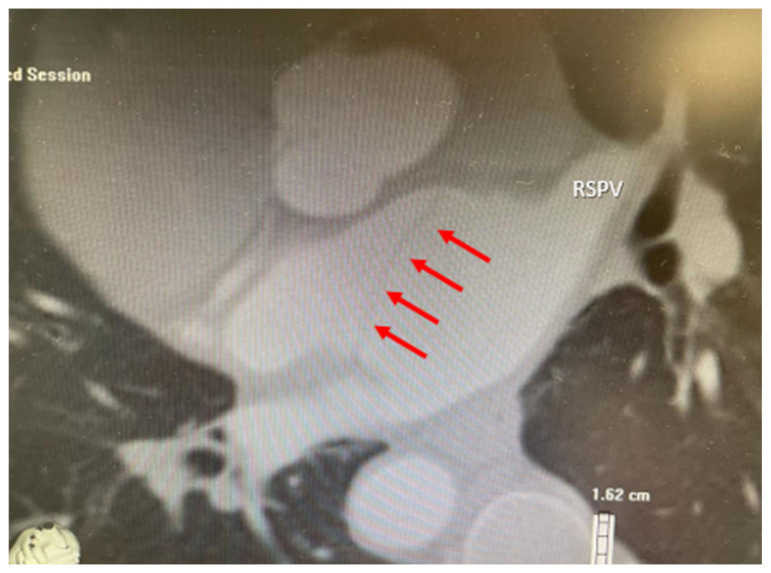
Multidetector computed tomography angiography confirming the presence of two separate left atrial chambers, the superoposterior atrial chamber which receives the pulmonary veins and the inferoanterior atrial chamber, separated by a membrane (red arrows). RSPV—right superior pulmonary vein.

An ECG on admission showed a normal sinus rhythm of 70 bpm. Transthoracic echocardiography revealed normal left ventricular function, mild mitral regurgitation and a mildly dilated left atrium, with a structure that resembled a membrane cutting transversely from left to right at this level. CTS was suspected, which was easily confirmed by transesophageal echocardiography (TEE) (Figure 1). TEE showed no significant gradient at the level of the membrane, suggesting a big fenestration between the superior and the inferior cavities of the left atrium. Multidetector computed tomography angiography further characterized the left atrial anatomy, showing a superoposterior atrial chamber in which a common left pulmonary vein (the superior and inferior left pulmonary veins drain into the LA through a common pulmonary venous ostium) and two separate right pulmonary veins were tributary and an inferoanterior chamber which contained the mitral valve and left atrial appendage. The two chambers were separated by a membrane inserted from the left ridge, in immediate proximity to the left common pulmonary vein, to the interatrial septum in its anterior region (Figure 2).

**Figure 3:**
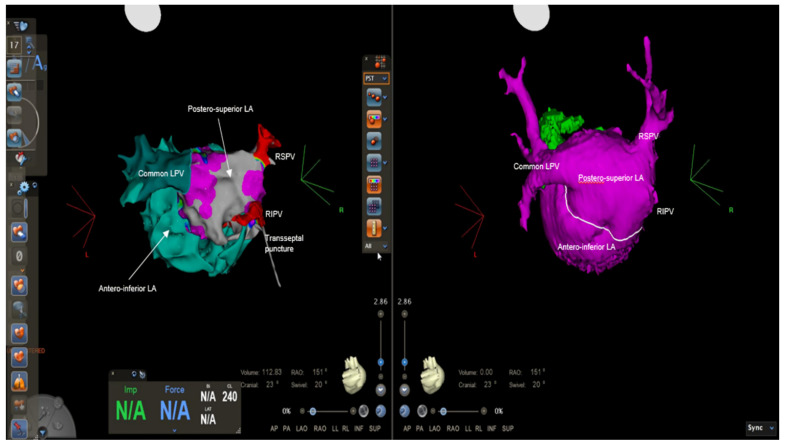
Electroanatomical mapping using CARTO 3 system and CT reconstruction showing the anatomy of the pulmonary veins: two separate pulmonary veins on the right and a common left pulmonary vein draining into the posterosuperior left-atrial chamber, where we can also notice the transseptal puncture. LA = left atrium, LPV = left pulmonary vein, RIPV = right inferior pulmonary vein, RSPV = right superior pulmonary vein.

**Figure 4:**
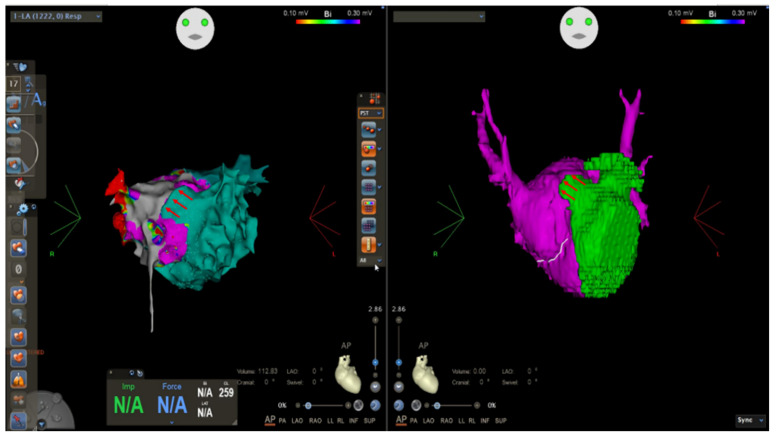
Electroanatomical mapping using CARTO 3 system and CT reconstruction showing anterior fenestration in the CTS membrane (red arrows).

We decided to perform AF catheter ablation under general anesthesia using live TEE guidance. Transseptal access to the superoposterior part of the left atrium (pulmonary chamber) was achieved. A bidirectional guiding sheath was introduced into the LA in order to gain better catheter stability (CARTO VIZIGO™ Bi-Directional Guiding Sheath; Biosense Webster, Inc., Diamond Bar, CA, USA). Electroanatomical mapping performed using a PentaRay NAV catheter and the CARTO 3 system (Biosense Webster, Inc., Diamond Bar, CA, USA) confirmed the common left pulmonary vein and two distinct pulmonary veins on the right, and no low-voltage areas were detected inside the left atrium, meaning no fibrosis was present (Figure 3). A large fenestration was seen during electroanatomical mapping, localized in the anterior part of the septum, which was not very clear until this moment (Figure 4).

We performed circumferential point-by-point high-power, short-duration (50 W, ablation index 450 on the anterior wall and 320 on the posterior wall) radiofrequency isolation of the left common pulmonary vein and right pulmonary veins using a SmartTouch SF force-sensing catheter (Biosense Webster, Inc., Diamond Bar, CA, USA). Catheter stability was lower on the left ridge, in the proximity of the CTS membrane, and there was an unusual catheter curve to the left ridge, but good contact force was finally achieved (>10 g) and the vein was successfully isolated. No extra ablation points were applied inside the left atrium. The isolation of the pulmonary veins was verified at the end of the procedure by both entrance- and exit-block. No complications appeared during and after the procedure and the patient was symptom-free at six months post-ablation, with 24 h ECG monitoring showing no AF recurrence.

## 3. Discussion

Cor triatriatum sinister is a rare congenital cardiac anomaly, most often diagnosed during childhood or infancy [1,2,3,4,5,6]. When it is diagnosed in adults, the most common presentation is for new onset atrial fibrillation [2,5,6,7]. Taking into account that most of these patients are in their young adulthood, catheter ablation can be considered as first line therapy [8].

CTS is a rare finding in the electrophysiology laboratory that could create difficulties during atrial fibrillation catheter ablation even for the most experienced electrophysiologists. A thorough imaging evaluation before performing the ablation for the purpose of an assessment of the exact anatomy of the left atrium and pulmonary veins is crucial in order to achieve good outcomes in terms of safety and efficacy [4,6,9,12,13]. Furthermore, electroanatomical mapping further defines the anatomy of important left atrial structures in patients with CTS during catheter ablation for atrial fibrillation [6].

The first case of atrial fibrillation ablation in a patient with CTS was reported by Yamada et al. in 2008 [14]. Since then, 14 other clinical cases were published, including one re-do by Yamada, focusing on the importance of anatomy in order to achieve a safe left atrial access through transseptal puncture to the posterosuperior atrial chamber [4,6,9,12,13,14,15,16,17,18,19,20]. However, neither of these reports focused on the impact of the anatomy of pulmonary veins on catheter manipulation and procedure outcome.

In this case, the left common pulmonary vein posed a challenge for catheter manipulation, as the CTS membrane inserted directly in its proximity, on the left ridge. While the left common pulmonary vein is a common anatomical variant in patients undergoing AF catheter ablation [10,11], there are no reports of this particular anatomy in patients with CTS. Moreover, the left ridge is recognized as a region where the catheter has reduced contact with the tissue [21], with the presence of both the CTS membrane and left common pulmonary vein posing further challenges for successful isolation of pulmonary veins. Despite the fact that, in our patient, catheter stability was lower on the left ridge, given the unusual curve of the ablation catheter passing through the interatrial septum and directed towards the narrow ridge between the ostium of the left common pulmonary vein and the insertion of the CTS membrane, we achieved a good contact force (>10 g) in this region and performed the successful isolation of all pulmonary veins, including the left common pulmonary vein. However, it is noteworthy that the left atrial anatomy in such patients, including that of pulmonary veins and CTS membrane insertion, may pose a challenge in the context of obtaining good contact force and catheter stability.

Thirteen out of the 15 previous reported cases of AF ablation in patients with CTS do not describe the used power-duration approach [4,6,9,13,14,15,16,17,18,19,20]. The remaining two case reports include one patient for whom low-power, long-duration settings (25–30 W, 30 s) were used [12] and one cryoballoon ablation [6]. Considering that high-power, short-duration is a relatively new approach for AF catheter ablation [22,23,24] and only 2 out of these 13 cases were published after 2018 [4,9], the present case may be the first report of high-power, short-duration AF ablation in a CTS patient.

In studied populations, results on arrhythmia recurrence after atrial fibrillation catheter ablation in patients with the left common pulmonary vein variant are controversial [10,11,21,25]. Although there are studies that show that atrial fibrillation recurrence is higher in patients with the left common pulmonary vein variant [11], most studies show good outcomes in these patients [21,25]. In our case, the patient was free of atrial fibrillation 6 months after ablation.

As the majority of the literature reports recommend, transseptal puncture was performed to the posterosuperior atrial chamber, which gave us direct access to all pulmonary veins, including the left pulmonary common vein. However, in some of the published cases [4,14,17], the transseptal approach was performed into the anteroinferior atrial chamber, with the authors reporting challenges in terms of obtaining access to the pulmonary veins’ antra due to the passing of the catheters through the fenestration. Still, Yamada et al. reported that the ablation of the right inferior pulmonary vein was more challenging when left atrial cannulation was performed to the posterosuperior compared to anteroinferior chamber in the same re-do patient [14,15]. Nevertheless, access to the posterosuperior atrial chamber should be performed first in all patients [6], including patients with the left common pulmonary vein variant.

Tokuda et al. reported a case of atrial fibrillation catheter ablation in a CTS patient in which all pulmonary veins, including left and right veins, drained into the posterosuperior atrium through a total common trunk [16]. However, the anatomy of the pulmonary veins was different to that of to our patient, with ablation being performed by isolating the single total pulmonary trunk.

In our patient, there was no need for extra ablation sites inside the left atrium, as electroanatomical mapping showed no low-voltage areas and no electrical potentials on or adjacent to the membrane. Other studies reported electrical potentials on or around the membrane [9,16], and depending on the type of atrial fibrillation, the need of secondary sites of ablation [9,16,17,18,20], including left atrial roof line, box or mitral isthmus ablation.

Finally, although intracardiac echocardiography was used for live guidance during the procedure in most of the reported cases, for the purpose of transseptal puncture [4,6,9,12,13,16,18,20], transesophageal echocardiography was also used in several reports [17,19], including ours, without any disadvantages reported. Intraoperative imaging by ICE or TEE, together with electroanatomical mapping and aided by preoperative advanced imaging techniques such as multidetector computed tomography, is essential for describing the exact anatomy of the left atrium and pulmonary veins in CTS patients undergoing AF catheter ablation. All together, these imaging techniques enable electrophysiologists to plan and perform procedures more safely and efficiently.

## 4. Conclusions

AF is the most common presentation in adult patients with CTS. A thorough understanding of the anatomy of the left atrium, including that of the pulmonary veins, CTS membrane and fenestration, and interatrial septum allows electrophysiologists to perform AF catheter ablation safely and with good efficacy. The presence of a left common pulmonary vein in patients with CTS may pose challenges with respect to catheter manipulation, particularly on the left ridge where the membrane is inserted. Good contact force (>10 g) using a high-power, short-duration approach can result in a good procedural outcome in patients with CTS and the left common pulmonary vein variant.

## Data Availability

Not applicable.
